# Can We Predict the Pressure Induced Phase Transition of Urea? Application of Quantum Molecular Dynamics

**DOI:** 10.3390/molecules25071584

**Published:** 2020-03-30

**Authors:** Anna Mazurek, Łukasz Szeleszczuk, Dariusz Maciej Pisklak

**Affiliations:** Faculty of Pharmacy, Medical University of Warsaw, Chair and Department of Physical Pharmacy and Bioanalysis, Department of Physical Chemistry, Banacha 1 str., 02-093 Warsaw, Poland; annamazurek21@gmail.com (A.M.); dpisklak@wum.edu.pl (D.M.P.)

**Keywords:** CASTEP, urea, phase transition, quantum molecular dynamics, NPT, DFT, periodic calculations

## Abstract

Crystalline urea undergoes polymorphic phase transition induced by high pressure. Form I, which is the most stable form at normal conditions and Form IV, which is the most stable form at 3.10 GPa, not only crystallize in various crystal systems but also differ significantly in the unit cell dimensions. The aim of this study was to determine if it is possible to predict polymorphic phase transitions by optimizing Form I at high pressure and Form IV at low pressure. To achieve this aim, a large number of periodic density functional theory (DFT) calculations were performed using CASTEP. After geometry optimization of Form IV at 0 GPa Form I was obtained, performing energy minimization of Form I at high pressure did not result in Form IV. However, employing quantum molecular isothermal–isobaric (NPT) dynamics calculations enabled to accurately predict this high-pressure transformation. This study shows the potential of different approaches in predicting the polymorphic phase transition and points to the key factors that are necessary to achieve the success.

## 1. Introduction

### 1.1. Molecular Modeling of Pressure Induced Phase Transition

Polymorphism is a crucial phenomenon in many scientific disciplines, since the molecular packing determines the functional properties of organic solids. One of the methods that can be used to obtain new polymorphs is exposing the molecular crystals to high-pressure in order to induce the phase transition [1]. Many studies deal with the experimental pressure-induced polymorphic transformations in molecular solids [2,3,4], in some cases the high-pressure polymorph may have the same space group symmetry as the original ambient-pressure form and even be isostructural with it, which is defined as isosymmetric phase transition [5,6]. However, in most cases the polymorphic phase transition is accompanied with the change of not only cell dimensions but also the crystal space group.

Due to the fact that the high pressure studies are more demanding in terms of cost and time than experiments performed under normal pressure, it would be perfect if the DFT calculations could be used to accurately predict the influence of the high pressure on the crystal structure and stability. The source of difficulty associated with the use of computational tools to investigate the polymorphism in organic systems results from the requirement to describe both accurately and simultaneously the effects of covalent, ionic, hydrogen, and van der Waals interactions as their energies span over three orders of magnitude, from ca. 100 kcal/mol to 0.1 kcal/mol [7] while the energy differences between experimentally observed polymorphs are usually less than 1 kcal/mol per molecule but can even be lower than 1 kJ/mol [8]. Due to that, molecular mechanics calculations or force field based molecular dynamics calculations are in many cases not accurate enough to precisely predict the effect of pressure on organic solids.

Fortunately, calculations on molecular crystals using density functional theory (DFT) based programs that enable to include the periodic boundary conditions of a studied system and a planewave basis set, such as CASTEP [9], have proven to be very accurate. Still, in most of the reported studies on the relative stability of the polymorphic forms solely the lattice energies [10,11,12] or, in less number of cases, free energy differences [13,14] of the structures are being calculated and compared. While those computational studies are indeed very interesting and appreciated, since their results enable the insight into the structure and stability of polymorphic forms, accurate phase transition modeling—defined here as a possibility to predict the changes in the crystal structure when exposed to the high pressure—is a much more complicated and challenging task.

### 1.2. Polymorphism of Urea

Nowadays, four polymorphic phases of urea, named: I, III, IV, V are known [15]. Such numeration has a historical justification. In 1916 Birdgman reported about phase II appearing above 0.06 GPa and at T = 373 K [16] but in a given condition such a form has never again been achieved. It was only in 2019 when the phase II was reported to be coincidental with the phase IV [15]. On the contrary, the form V appears at much higher pressure, according to neutron diffraction or Raman data, respectively, above 7.20 GPa or 8.0 GPa and belongs to Pmcn crystal class [17]. However, so far unit cell parameters have been reported only for phases I, III and IV, thus in this work only these phases have been considered.

Form I belongs to a crystallographic tetragonal system (unit cell parameters: a = back, α = β = γ = 90°), space group P4̅2_1_m. Phases III and IV (unit cell parameters: a ≠ b ≠ c, α = β= γ = 90°), both orthorhombic system, have been parameterized, respectively, as P2_1_2_1_2_1_ and P2_1_2_1_2 space group. In one-unit cell of Forms I and IV there are two urea molecules (Z = 2) whereas in Form III four molecules (Z = 4).

At 296 K polymorphic transition of Form I to Form III occurs at 0.48 GPa, whereas between Form I and III at about 2.8 GPa. It has been proven multiple times that at the normal conditions Form I is the most stable one while at the increased pressure, c.a. 3.10 GPa, Form III is the only stable polymorph [15].

The tetragonal structure of Form I has been confirmed down to 12K [18]. In this crystal structure, one carbonyl group is the acceptor of four N-H^…^O hydrogen bonds, which is very unusual. Moreover, the planar and C_2V_ symmetric urea molecule that exists in Form I is energetically less stable than C_2_ and Cs symmetric conformations with the NH_2_ groups twisted off from the molecular plane, as observed by microwave spectroscopy [19]. The metastable molecular conformation and the voids present in Form I are prerequisites for the high susceptibility of the structure to elevated pressure. The hydrogen bonds in crystalline urea undergo a considerable strengthening upon compression, which was confirmed by the softening of the vibrational modes involving the N-H groups [20]. A very detailed knowledge of urea polymorphic structures enables to compare the experimental and computational results [21] and gives the chance to accurately optimize the calculation methodology that would also be suitable for other organic polymorphs. Therefore, using urea as a model compound, we have decided to conduct the computational study in order to explore the possible application of periodic DFT calculations in predicting the pressure induced polymorphic phase transition.

For several reasons, in this study particular attention was put on the transition between Form I and IV, though at normal temperature those two Forms are separated by the orthorhombic Form III First, the Form III was postulated to be stable only up to 370 K, while Form I and Form IV are stable even at higher temperatures. Further, the large energetic barrier separating Forms III and IV is highlighted by the huge metastability P-T region of Form III [15]. The thermodynamic boundary of this phase at ambient temperature is close to 1.3 GPa, however the pressures at which the Form I to Form III and Form III to Form IV transitions occur are strongly temperature-dependent. Since, to the best of our knowledge, this study is the first in which the quantum molecular dynamics was applied to study the phase transition of urea, it was a safer option to study the transitions between the forms that are the only stable ones at the studied pressures, regardless of the temperature (Form I at 0 GPa and Form IV at 3.10 GPa). Especially, since in the molecular dynamics calculations some fluctuations of temperature occur.

The first aim of this study was to evaluate the accuracy of the crystal structure optimization of polymorphic forms of urea at normal and increased pressure. The next goal was to check if such calculations can be used to correctly predict the relative stability of the studied forms by comparing the energy and free energy values. Finally, using the optimized methodology, we wanted to determine if it is possible to foresee the pressure induced polymorphic phase transitions, that is to obtain the new polymorphic form of urea while starting from the other form and applying the appropriate pressure during calculations. Knowledge of the strengths and limitations of such an approach is necessary for the development of modern crystal engineering [22].

## 2. Results

### 2.1. Optimization of the Calculations Method

The very first aim of this work was to optimize the calculations method, including parameters listed in the calculations methodology section. To achieve this, a series of geometry optimization calculations were performed, including the optimization of the unit cell parameters. The calculations presented in this Section 2.1 were performed at the pressure values at which the studied forms are stable, that is 0 GPa for Form I and 3.1 GPa for Form IV. For the more convenient assessment of the accuracy of calculations and influence of the tested parameters on the results, the obtained values were presented in tables together with the corresponding experimental ones (crystal structures UREAXX12 for Form I and UREAXX26 for Form IV.) Though no symmetry requirements resulting from the crystal space group were applied, the change of the crystal space group has not been observed in any of the calculations results described here in Section 2.1. Further, though the unit cell angles were not fixed, their values have not changed during the optimization and have remained constant (90^o^).

#### 2.1.1. Influence of the Choice of Functional and Dispersion Correction on the Calculations Accuracy

In the first set of calculations the ultra-fine CASTEP standards regarding SCF convergence criteria, 3 × 3 × 3 (for Form I) and 4 × 2 × 3 (for Form IV) Monkhorst–Pack k-point grid, 48 × 48 × 40 (for Form I) and 30 × 60 × 40 (for Form IV) FFT grid were used. The on the fly generated (OTFG) NCP were generated using Koelling–Harmon (KH) relativistic treatment and E_cut_ of 990 eV, with the variables being the choice of the DFT functional and applied dispersion correction method during geometry optimization. The calculation results for Form I and Form IV can be found in the Table 1 and Table 2, respectively.

Analysis of the results from Table 1; Table 2 shows that the choice of functional has a huge influence on the final unit cell dimensions. In some cases, i.e., HSE06 and sX for Form I, the obtained volumes differ from each other almost three times. Though in the literature there are many examples in which each of the functional tested here was the most accurate one [23], proving its usefulness in those particular cases, it is also well known that the results using different functionals may differ significantly. Therefore, it is always the best option to test various functionals and choose the one that provides the most accurate results. The data presented above indicate, that in the case of crystalline urea the best options are GGA PBESOL, GGA PBE TS and GGA WC. Those functionals provided the most accurate results for both Form I and Form IV and were chosen for further calculations.

It is also worth to notice that even using the functionals for which the less accurate results were obtained, some universal trends could be observed, i.e., the changes in the unit cell dimensions, when compared with the experimental ones, were common for all of the cell lengths, resulting in either increase (GGA PBE, GGA RPBE, GGA PW91) or decrease (LDA CA-PZ, GGA PW91 OBS) of all three of them (a, b, c), with an exception for nonlocal potentials (HSE03, HSE06).

#### 2.1.2. Energy Cutoff Value Optimization

One of the most important parameters determining the accuracy and computational cost of a CASTEP calculation is the size of the basis set, defined by the energy cutoff value (E_cut_). The truncation of the basis set at a finite E_cut_ leads to an error in the computed values. The straightforward and most efficient way to reduce the magnitude of this error is to systematically increase the value of the E_cut_, up to until the calculated values converge within the required tolerance. In the CASTEP calculations the values of the E_cut_ that correspond to the coarse, medium, fine and ultra-fine settings are element specific and are taken from the pseudopotential files stored in the database. These particular values were determined from the series of convergence tests, including geometry optimization and energy calculations, mostly for single atoms and diatomic molecules. Since the elements differ in their E_cut_, the highest value for the selected quality setting among the elements present in the structure is accepted, which in the case of urea was the oxygen.

To optimize the energy cutoff value four geometry optimization calculations at 0 GPa with increasing value of E_cut_ were performed, using Form I as a studied structure and PBESOL as a functional. The results can be found in Table 3.

In the studied case, the E_cut_ of 898 eV could probably be sufficient taking into consideration solely the unit cell dimensions. However, to ensure that underestimation of E_cut_ will not have an influence on the accuracy of further calculations, finally the 990 eV had been chosen as a value that would be optimal, in terms of both computational cost and accuracy. The differences between the energy values obtain using 990 and 1200 eV were found to be negligible (lower than 10^−3^ eV).

#### 2.1.3. Electronic Options Optimization

In this study particular focus was put on the optimization of electronic options, above all because the geometry optimization proved later to be unsuccessful in predicting the Form I to Form IV transformation at 3.10 GPa, which will be described in details in Section 2.2.2. Since the quantum molecular dynamics calculations are significantly more computationally demanding, in comparison with geometry optimization, therefore any saving of computational time resulting from not overestimating the calculations quality criteria would be especially beneficial. Finally, as in most cases, it was important to ensure that the possible inconsistencies between the experimentally obtained and calculated values or possible failures would not result from the calculations quality reasons.

The k-point set used in a calculation defines the accuracy of the Brillouin zone sampling. The magnitude of error in the total energy due to limited k-point sampling can be reduced by using a denser set of k-points, in similar way as the convergence with respect to E_cut_ is achieved. In this work the seven options of the Monkhorst-Pack discrete k-points sets, uniform in each reciprocal space direction, were tested: 1 × 1 × 2, 2 × 2 × 2, 2 × 2 × 3, 3 × 3 × 3, 3 × 3 × 4, 4 × 4 × 4 and 4 × 4 × 5. Those sets correspond to the following separation values (Å^−1^) 0.12, 0.09, 0.08, 0.07, 0.06, 0.05 and 0.04. The results of such convergence test are presented in Table 4.

In the studied case, the separation value of 0.07 Å^−1^ was found to be the converged one. Again, as in the E_cut_ optimization, the application of separation of 0.08 Å^−1^ would probably be enough, however as this parameter does not increase the time of calculations as much as the increase of E_cut_, we have decided to choose the 0.07 Å^−1^ for any further calculations.

#### 2.1.4. Supercell Approach

So far only the geometry optimization calculations have been discussed. However, as described in the introduction, to determine the relative stability of polymorphs, the free energy (ΔF) values should be compared, which can be obtained via the phonon properties calculations. However, if phonon calculations are performed by using finite differences, as in this study, a large enough cell has to be used so that the effect of one atomic displacement does not produce artifacts between periodic images. To ensure that the cell dimensions are large enough to obtain the accurate thermodynamics results the calculations using 2 × 2 × 2 and 1 × 1 × 2 supercells of Form I were performed. The obtained results were compared with the ones received for a unit cell (1 × 1 × 1) and can be found in the Table 5. Those calculations were performed using previously optimized values of E_cut_ (990 eV), k-point separation (0.07 Å^−1^) employing PBESOL functional.

The analysis of the results presented in the Table 5 showed that employing the supercells for the calculation did not have an influence on the received optimized unit cell dimensions nor on the energy and thermodynamics values in conversion to unit cell.

### 2.2. Prediction of the Phase Transition – Geometry Optimization and Thermodynamic Calculations Approach

#### 2.2.1. Form IV to Form I Transition at 0 GPa

Having optimized the calculations method, it was then possible to proceed to the main aim of this study which was the desire to observe the phase transitions of Form IV to Form I at low pressure and from Form I to Form IV at high pressure.

Since the low pressure polymorph of urea has been very well studied and proved multiple times to be the most stable form at 0 GPa, we have decided to start with modeling the Form IV to Form I phase transition. To begin with, the simple method of Form IV geometry optimization at 0 GPa, without any constrains resulting from space group symmetry, has been chosen. A series of calculations using previously optimized values of E_cut_ (990 eV), k-point separation (0.07 Å^−1^) employing GGA PBESOL, GGA PBE TS and GGA WC functionals have been performed. Those three functionals have been previously (Section 2.1.1.) proved to be the most accurate in modeling the crystalline urea. For comparison, exactly the same calculations have been done for the Form I. The results can be found in Table 6.

Analysis of the results presented in Table 6 proved that this aim has been successfully accomplished. Though the accuracy of the obtained results depends on the chosen functional, in all three approaches the correct change of the crystal space group was observed as well as final unit cell dimensions obtained using Form IV as the initial structure match those obtained using Form I as initial structure, with good accuracy. Additionally, almost exact energy and free energy values obtained using the same functional but starting from different initial structure proves the usefulness of this approach. It is worth to notice here that the experimental crystal structure is always an average of the slightly different conformations that occur under specified temperature. Therefore, it is not surprising that using the same functional two slightly different structures possessing the same energy are obtained, suggesting the possible equilibrium of those under experimental conditions.

**Figure 1:**
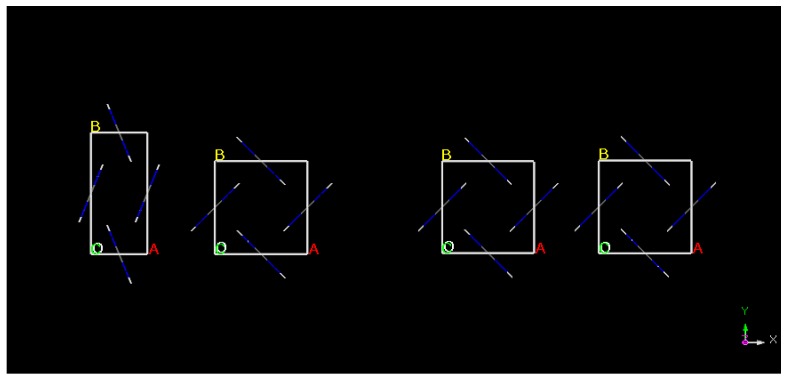
Structures of (starting from left) Form IV before optimization, Form IV after optimization at 0 GPa, Form I before optimization and Form I after optimization at 0 GPa.

To conclude, by performing geometry optimization calculations we have predicted the Form I is the more stable form at 0 GPa and starting from the less stable one (Form IV) we have obtained the more stable one with good accuracy. To better visualize the changes in the unit cell dimensions that occur as a result of geometry optimization crystal structures were presented on Figure 1.

#### 2.2.2. Form I to Form IV Transition at 3.10 GPa

Encouraged by the success described in Section 2.2.1, an attempt has been made to repeat the same procedure, that is geometry optimization starting from Form I and IV, but this time at 3.10 GPa. A series of similar calculations, without any constrains resulting from space group symmetry, using previously optimized values of E_cut_ (990 eV), k-point separation (0.07 Å^−1^) employing GGA PBESOL, GGA PBE TS and GGA WC functionals have been performed on both Form I and Form IV. The results can be found in Table 7.

Analysis of the results presented in Table 7 indicates, that this time geometry optimization procedure was not sufficient to obtain the more stable Form IV, starting from the less stable one—Form I, and applying pressure of 3.10 GPa during the calculations. Regardless of the potential used, while starting from the Form I the space group has been preserved and the final structure differed significantly from the one obtained using Form IV as the initial one. During the geometry optimization of Form I only the compression effect was observed that manifested itself with the decrease of unit cell dimensions and volume. This could suggest that transformation of Form I to Form IV at 3.10 GPa is connected with crossing the energy barrier required for the change of the space group that couldn’t be overpassed during geometry optimization. A solution to this problem, quantum molecular dynamics (QMD), was performed and described in Section 2.3.

However, two observations were optimistic. First, the accuracy of the calculations, while starting from Form IV, was very good. Second, the positive values of differences in the E and F between the Form I and Form IV proved that using geometry optimization we can correctly predict that at 3.10 GPa Form IV is the more stable one. This is in accordance with experimental results as Form I has never been observed at pressure higher than 0.47 GPa. However, while correct prediction of the order of stability is a success, the aim of this study was even more ambitious and consisted of accurate crystal structure prediction. Since no more options were left at this point, as the method was neatly optimized and multiple functional have been checked, we have decided to perform more demanding but promising quantum molecular dynamics calculations.

### 2.3. Prediction of the Phase Transition—Quantum Molecular Dynamics Approach

**Figure 2:**
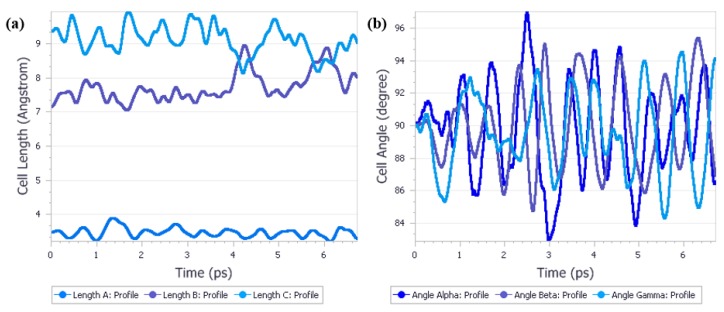
Quantum molecular dynamics calculations results on Form IV at 3.10 GPa. (**a**) Cell length analysis; (**b**) Cell Angle analysis.

Before performing the calculations on the Form I, we have decided that the first object of QMD calculations would be Form IV. This simulation has been done for several reasons. First, we wanted to confirm that under those calculations conditions no other phase transition would occur. Though experimentally Form IV was found to be the only stable one to exist at 3.10 GPa, we wanted to ensure that the results of calculations would confirm this experimental observation. Secondly, since introduction of the kinetic energy associated with the temperature always results in the structural parameters fluctuations, it was necessary to determine the magnitude of such fluctuations. Last but not least, since the geometry optimization has been done at 0 K while in the molecular dynamics the 298 K value has been set, we wanted to observe how the thermal expansion would affect the unit cell dimensions. Additionally, to increase the number of molecules in the unit cell and therefore to increase the likelihood of the phase transition by increasing the number of possible arrangements within the cell, we have decided to perform the MD calculations using 1 × 1 × 2 supercell approach, that is by adding one unit cell along the axis c. To achieve those goals, molecular dynamics calculations using isothermal–isobaric (NPT) ensemble were performed on Form IV at 3.10 GPa and the results were presented on Figure 2.

Analysis of the results of this QMD calculations provided answers to the questions stated above. First, no phase transition was observed during the time of simulation, which is in agreement with experimental results. Second, some fluctuations for the unit cell dimensions were observed, however they did not exceed ± 0.8 Å for a and b, ± 0.4 Å for c and ± 5 ° for cell angles. Finally, the systematic changes in the unit cell lengths caused by thermal motions were found to be at the same level of magnitude as the thermal fluctuations.

**Figure 3:**
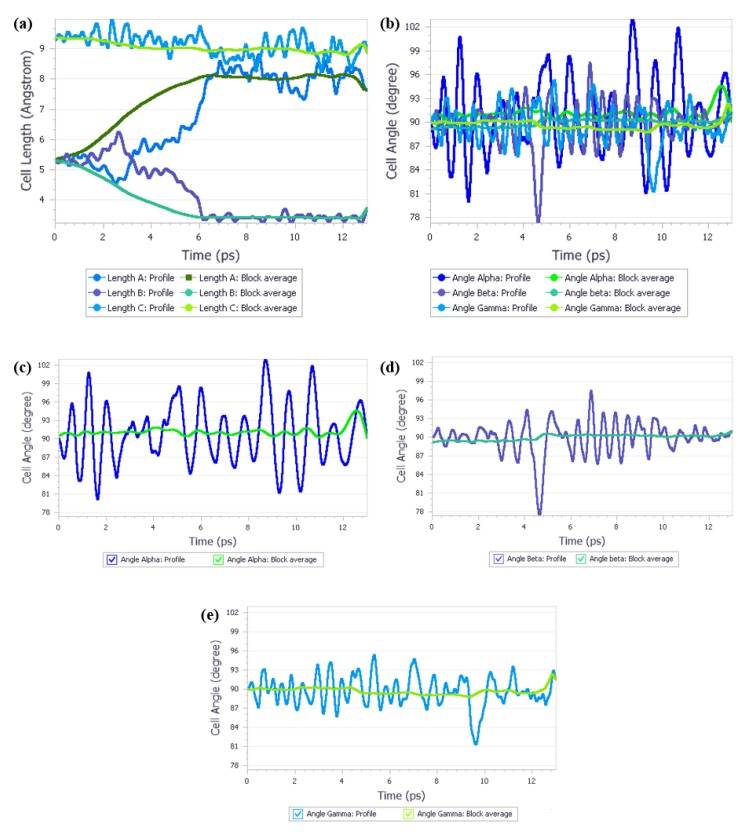
Quantum molecular dynamics calculations on Form I at 3.10 GPa. Block average was calculated over 5 ps simulation time. For better view cell angle analysis was split into three separate charts. (**a**) Cell length analysis; (**b**) Cell angle analysis; (**c**) Angle Alpha; (**d**)Angle beta; (**e**) Angle gamma.

The next and final step of this project was performing the NPT QMD calculations at 3.10 GPa, starting from Form I. The parameters of those calculations, such as the time step or E_cut_ or functional were exactly the same as the ones for Form IV, with the only exception being the initial structure used for calculations. The results of this QMD can be found on Figure 3 and in Supplementary Materials Video S1.

As can be seen on the Figure 3, when the QMD calculations at 3.10 GPa were performed starting from the Form I, substantial changes in the unit cell lengths were observed after c.a. 6 ps. During the first 6 ps of calculations Form I preserved its shape, though some fluctuations caused by thermal motions could be observed, similarly as for the Form IV. Since in the Form I lengths “a” and “b” are equal, which is the requirement of the tetragonal crystal system that this form belongs to, a noticeable increase of the “b” length that occurred at c.a. 2.5 ps was simultaneously compensated by the decrease of the “a” length, resulting in the crystal preserving its volume. During 3.0–5.0 ps simulation time a small difference between the “a” and “b” lengths could be observed. Suddenly, after 5 ps, a strong trend in the unit cell lengths “a” and “b” changes emerged which lasted 1.5 ps. After total 6.5 ps of simulation the unit cell lengths reached their final values and since that moment only the thermal fluctuations were observed. As anticipated, the final unit cell lengths equaled those received for the Form IV and the final space group was determined as P 21 21 21. Therefore, it is justified to state that by applying properly set QMD calculations it was possible to predict the phase transition of crystalline urea and accurately determine the unit cell lengths and space group of the most stable form at the conditions specified in the calculations.

## 3. Materials and Methods

The density functional theory (DFT) calculations of geometry optimization, dynamics and phonon properties were carried out with the CASTEP program [9] implemented in the Materials Studio 2017 software [24] using the plane wave pseudopotential formalism.

On the fly generated (OTFG) norm conserving pseudopotentials (NCP) were generated using Koelling–Harmon (KH) scalar relativistic approach [25].

The Perdew–Burke–Ernzerhof (PBE) [26] pure or with Tkatchenko–Scheffler (TS) [27] dispersion correction, Perdew–Wang (PW91) [28] pure or with Ortmann–Bechstedt–Schmidt (OBS) [29] dispersion correction, revised Perdew–Burke–Ernzerhof (RPBE) [30], Wu–Cohen (WC) [31], solid-design version of the PBE (PBESOL) [32] exchange-correlation functionals, defined within the generalized gradient approximation (GGA) as well as the local exchange-correlation functional of Perdew and Zunger [33] with the parameterization of the numerical results of Ceperley and Alder [34] (LDA CA-PZ), with or without the OBS method of dispersion correction and nonlocal potentials such as HF, HF-LDA, sX, sX-LDA, PBE0, B3LYP, HSE03 and HSE06 were used in the calculations.

Geometry optimization was carried out using the Broyden−Fletcher−Goldfarb−Shanno (BFGS) [35] optimization scheme and smart method for finite basis set correction. The parameters such as kinetic energy cutoff for the plane waves (E_cut_) and number of Monkhorst–Pack k-points during sampling for a primitive cell Brillouin zone integration [36] were optimized and discussed in this manuscript.

The experimental X-ray structure of urea Form I (refcode UREAXX12) and Form IV (UREAXX26) from the Cambridge Structure Database (CSD) were used as initial for calculations. During geometry optimization all atoms positions and the cell parameters were optimized, with no constraints resulting from the crystal space group that the studied forms belong to.

Born–Oppenheimer quantum molecular dynamics (QMD) [37] simulations were run in CASTEP using an NPT ensemble maintained at a constant temperature of 295 K using a Nosé thermostat Parinello barostat. For the QMD calculations the norm conserving pseudopotentials (NCP) were generated using the PBE TS functional and KH relativistic treatment. The kinetic energy cutoff for the plane waves (E_cut_) was set to 990 eV and the integration time step was set to 0.5 fs. No symmetry constraints were applied during the simulations.

The linear response density functional perturbation theory [38] (DFPT) implemented in the CASTEP code was used to obtain the phonon dispersion curves and phonon density of states from which thermodynamic quantities in the quasi-harmonic approximation: free energies (∆G), enthalpies (∆H) and entropies (∆S), at 295 K were evaluated.

To check the accuracy of computations the GGA PBE TS geometry optimization calculations of Form I at 0 GPa, starting from the experimental structure, were performed in triplicate. The results of those calculations can be found in Table 8. The difference in the calculated energies were found to be lower than 1 × 10^−3^ eV.

## 4. Conclusions

In this work the multiple DFT calculations on crystalline urea has been performed and analyzed using CASTEP program. The influence of the electronic parameters and applied functional on the results of calculations was evaluated in details. Using geometry optimization with no constraints resulting from the crystal space group applied it was possible to predict the Form IV to Form I transition at low pressure. However, this procedure was unsuccessful for the prediction of Form I to Form IV transition at 3.10 GPa. Nevertheless, this pressure induced polymorphic transitions was correctly predicted using NPT quantum molecular dynamics calculations.

This work shows that periodic DFT calculations can be successfully applied to study the phenomenon of polymorphic phase transitions and for accurate crystal structure prediction at normal and increased pressure. Such calculations may be very helpful in prediction of new polymorphic forms and planning of the high pressure crystal studies as well as in the interpretation of experimental results.

## Figures and Tables

**Table 1:** Results of geometry optimization for Form I at 0 GPa using various functionals.

	a [Å]	b [Å]	c [Å]	Volume [Å^3^]	Energy [eV]
HSE06	4.013	4.013	5.965	96.04	−2424.267
HSE03	4.121	4.121	5.900	100.21	−2439.177
LDA CA-PZ OBS	5.030	5.030	4.505	113.96	−2430.941
GGA PW91 OBS	5.267	5.267	4.632	128.51	−2434.915
LDA CA-PZ	5.333	5.333	4.550	129.41	−2428.225
SX-LDA-CA-PZ	5.464	5.464	4.572	136.51	−2378.664
GGA PBE Grimme	5.464	5.464	4.679	139.68	−2430.066
GGA PBESOL	5.559	5.559	4.628	143.00	−2422.631
**Experimental**	5.565	5.565	4.684	145.06	
GGA PBE TS	5.573	5.573	4.706	146.17	−2430.001
GGA WC	5.623	5.623	4.628	146.32	−2427.256
GGA PW91	5.813	5.813	4.706	159.03	−2432.707
GGA PBE	5.807	5.807	4.718	159.12	−2429.205
GGA RPBE	6.292	6.292	4.846	191.83	−2428.408
sX	7.419	7.419	5.218	287.19	−2042.342

**Table 2:** Results of geometry optimization for Form IV at 3.10 GPa using various functionals.

	a [Å]	b [Å]	c [Å]	Volume [Å^3^]	Energy [eV]
HSE06	2.771	6.998	4.439	86.08	−2422.8993
HSE03	2.826	7.022	4.471	88.74	−2437.6195
LDA CA-PZ OBS	3.016	6.987	4.449	93.76	−2429.3839
GGA PW91 OBS	3.211	7.138	4.585	105.08	−2432.8730
LDA CA-PZ	3.344	7.001	4.547	106.45	−2425.9929
SX-LDA-CA-PZ	3.476	7.021	4.559	111.27	−2405.4867
GGA PBE Grimme	3.345	7.264	4.646	112.91	−2427.7257
GGA PBESOL	3.523	7.047	4.619	114.68	−2420.1431
GGA WC	3.545	7.028	4.617	115.03	−2424.7312
**Experimental**	3.414	7.360	4.606	115.74	
GGA PBE TS	3.474	7.166	4.683	116.56	−2427.5739
GGA PW91	3.742	7.059	4.693	123.95	−2429.9929
GGA PBE	3.746	7.069	4.704	124.58	−2426.4854
GGA RPBE	4.110	7.003	4.806	138.31	−2425.3566
sX	4.066	7.997	5.053	164.33	−2038.5904

**Table 3:** Results of CASTEP calculations for Form I at 0 GPa, the convergence of the unit cell dimensions and energy with respect to the energy cutoff for the plane waves E_cut_.

E_cut_ [eV]	a [Å]	b [Å]	c [Å]	V [Å^3^]	E [eV]
789.1	5.604	5.604	4.660	146.34	−2420.839
898	5.566	5.566	4.640	143.74	−2422.248
990	5.559	5.559	4.629	143.04	−2422.631
1200	5.558	5.558	4.619	142.69	−2422.634

**Table 4:** Results of CASTEP calculations for Form I at 0 GPa, the convergence of the unit cell dimensions and energy with respect to the Monkhorst–Pack k-point set and separation.

k-Point Set	k-Point Separation [Å^−1^]	a [Å]	b [Å]	c [Å]	V [Å^3^]	E [eV]
1 × 1 × 2	0.12	5.329	5.329	4.670	132.60	−2422.879
2 × 2 × 2	0.09	5.562	5.562	4.606	142.50	−2422.676
2 × 2 × 3	0.08	5.558	5.558	4.629	143.00	−2422.632
3 × 3 × 3	0.07	5.559	5.559	4.629	143.04	−2422.631
3 × 3 × 4	0.06	5.559	5.559	4.631	143.11	−2422.631
4 × 4 × 4	0.05	5.557	5.557	4.629	142.94	−2422.632
4 × 4 × 5	0.04	5.558	5.558	4.630	143.02	−2422.631

**Table 5:** Results of CASTEP calculations for Form I at 0 GPa obtained using supercell approach. For more convenient comparison with the results obtained for the unit cell, some values have been divided. * ”c”, “V”, “E”, “F” divided by 2; ** ”a”, “b”, ”c” divided by 2, “V”, “E” “F” divided by 8.

Supercell	a [Å]	b [Å]	c [Å]	V [Å^3^]	E [eV]	F [eV]
Experimental	5.565	5.565	4.684	145.06		
1 × 1 × 1	5.559	5.559	4.629	143.04	−2422.631	−2419.464
1 × 1 × 2	5.560	5.560	9.260	286.25	−4845.263	−4838.919
1 × 1 × 2 *	5.560	5.560	4.630	143.13	−2422.631	−2419.459
2 × 2 × 2	11.125	11.125	9.260	1146.06	−19381.043	−19355.623
2 × 2 × 2 **	5.562	5.562	4.630	143.26	−2422.630	−2419.453

**Table 6:** Results of CASTEP calculations for Form I and Form IV at 0 GPa.

Initial Structure		a [Å]	b [Å]	c [Å]	V [Å^3^]	Space Group	E [eV]	F [eV]
Form I	Experimental (at 0 GPa)	5.565	5.565	4.684	145.06	P 4̅2_1_m		
	GGA PBESOL	5.559	5.559	4.629	143.04	P 4̅2_1_m	−2422.631	−2419.464
	GGA PBE TS	5.567	5.567	4.709	145.92	P 4̅2_1_m	−2430.001	−2426.788
	GGA WC	5.615	5.615	4.822	152.06	P 4̅2_1_m	−2427.257	−2424.099
Form IV	Experimental (at 3.10 GPa)	3.414	7.360	4.606	115.74	P2_1_2_1_2_1_		
	GGA PBESOL	5.635	5.505	4.632	143.68	P 4̅2_1_m	−2 422.631	−2419.469
	GGA PBE TS	5.303	5.806	4.709	144.97	P 4̅2_1_m	−2429.999	−2426.804
	GGA WC	5.606	5.631	4.629	146.13	P 4̅2_1_m	−2427.257	−2424.097

**Table 7:** Results of CASTEP calculations for Form I and Form IV at 3.10 GPa.

Initial Structure		a [Å]	b [Å]	c [Å]	V [Å^3^]	Space Group	E [eV]	F [eV]
Form I	Experimental (at 0 GPa)	5.565	5.565	4.684	145.06	P 4̅2_1_m		
	GGA PBESOL	5.237	5.237	4.574	125.47	P 4̅2_1_m	−2420.069	−2416.303
	GGA PBE TS	5.282	5.282	4.649	129.68	P 4̅2_1_m	−2427.370	−2423.576
	GGA WC	5.250	5.250	4.569	125.93	P 4̅2_1_m	−2424.673	−2420.898
Form IV	Experimental (at 3.10 GPa)	3.414	7.360	4.606	115.74	P2_1_2_1_2_1_		
	GGA PBESOL	3.523	7.047	4.619	114.68	P2_1_2_1_2_1_	−2420.143	−2416.369
	GGA PBE TS	3.474	7.166	4.683	116.56	P2_1_2_1_2_1_	−2427.574	−2423.764
	GGA WC	3.545	7.028	4.617	115.03	P2_1_2_1_2_1_	−2424.731	−2420.948

**Table 8:** Assessment of accuracy based on the results of geometry optimization for Form I at 0 GPa using GGA PBE TS functional repeated three times.

Number of Calculation	a [Å]	b [Å]	c [Å]	Volume [Å^3^]	Energy [eV]
1.	5.573	5.573	4.706	146.17	−2430.00091
2.	5,566	5,566	4.709	145.89	−2430.00127
3.	5.568	5.813	4.706	145.90	−2430.00137

## Supplementary Materials

The following are available online: Video S1: Molecular dynamics simulation of Form I at 4.0 GPa.

## References

[1] Fabbiani F., Allan R.D., David W., Moggach S., Parsons S., Pulham R. (2004). High-pressure recrystallisation—A route to new polymorphs and solvates. CrystEngComm.

[2] Yang L., Dai L., Li H., Hu H., Hong M., Zhang X. (2020). The Phase Transition and Dehydration in Epsomite under High Temperature and High Pressure. Crystals.

[3] Manjón F.J., Sans Tresserras J.A., Ibáñez J., Pereira A.L.J. (2019). Pressure-Induced Phase Transitions in Sesquioxides. Crystals.

[4] Boldyreva E. (2007). High-Pressure Polymorphs of Molecular Solids:  When Are They Formed, and When Are They Not? Some Examples of the Role of Kinetic Control. Cryst. Growth Des..

[5] Clarke S.M., Steele B.A., Kroonblawd M.P., Zhang D., Kuo I.-F.W., Stavrou E. (2020). An Isosymmetric High-Pressure Phase Transition in α-Glycylglycine: A Combined Experimental and Theoretical Study. J. Phys. Chem. B..

[6] Bull C.L., Funnell N.P., Ridley C.J., Pulham C.R., Coster P.L., Tellam J.P., Marshall W.G. (2019). Pressure-induced isosymmetric phase transition in biurea. CrystEngComm.

[7] Krzystyniak M., Drużbicki K., Romanelli G., Gutmann M.J., Rudić S., Imberti S., Fernandez-Alonso F. (2017). The road to a station for epithermal and thermal neutron analysis. Phys. Chem. Chem. Phys..

[8] Hoja J., Reilly A.M., Tkatchenko A. (2017). First-principles modeling of molecular crystals: Structures and stabilities, temperature and pressure. WIREs Comput. Mol. Sci..

[9] Clark S.J., Segall M.D., Pickard C.J., Hasnip P.J., Probert M.J., Refson K., Payne M.C. (2005). First principles methods using CASTEP. Z. Krist. Cryst. Mater.

[10] Szeleszczuk Ł., Pisklak D.M., Zielińska-Pisklak M. (2018). Does the choice of the crystal structure influence the results of the periodic DFT calculations? A case of glycine alpha polymorph GIPAW NMR parameters computations. J. Comput Chem..

[11] Sainz-Díaz C.I., Francisco-Márquez M., Soriano-Correa C. (2018). Polymorphism, Intermolecular Interactions, and Spectroscopic Properties in Crystal Structures of Sulfonamides. J. Pharm Sci..

[12] Sokal A., Pindelska E., Szeleszczuk Ł., Kołodziejski W. (2017). Pharmaceutical properties of two ethenzamide-gentisic acid cocrystal polymorphs: Drug release profiles, spectroscopic studies and theoretical calculations. Int. J. Pharm..

[13] Szeleszczuk Ł., Pisklak D.M., Zielińska-Pisklak M. (2018). Can we predict the structure and stability of molecular crystals under increased pressure? First-principles study of glycine phase transitions. J. Comput. Chem..

[14] Szeleszczuk Ł., Pisklak D.M., Gubica T., Matjakowska K., Kaźmierski S., Zielińska-Pisklak M. (2019). Application of combined solid-state NMR and DFT calculations for the study of piracetam polymorphism. Solid State Nucl. Magn. Reson..

[15] Dziubek K., Citroni M., Fanetti S., Cairns B.A., Bini R. (2017). High-Pressure High-Temperature Structural Properties of Urea. J. Phys. Chem. C.

[16] Bridgman P.W. (1916). Polymorphism at High Pressures. Proc. Am. Acad. ArtsSci..

[17] Weber H.P., Marshall W.G., Dmitriev V. (2002). High-Pressure Polymorphism in Deuterated Urea. Acta Cryst. A.

[18] Swaminathan S., Craven B.M., McMullan R.K. (1984). Theoretical and Experimental Studies of the Charge Density of Urea. Acta Crystallogr. Sect. B.

[19] Olejniczak A., Ostrowska K.A. (2009). H-Bond Breaking in High-Pressure Urea. J. Phys. Chem. C.

[20] Fanetti S., Citroni M., Dziubek K., Nobrega M.M., Bini R. (2018). The role of H-bond in the high-pressure chemistry of model molecules. J. Phys. Cond. Matter.

[21] Moses Abraham B., Adivaiah B., Vaitheeswaran G. (2019). Microscopic origin of pressure-induced phase-transitions in urea: A detailed investigation through first principles calculations. Phys. Chem. Chem. Phys..

[22] Rychkov D.A. (2020). A Short Review of Current Computational Concepts for High-Pressure Phase Transition Studies in Molecular Crystals. Crystals.

[23] Mardirossian N., Head-Gordon M. (2017). Thirty years of density functional theory in computational chemistry: An overview and extensive assessment of 200 density functionals. Mol. Phys..

[24] BIOVIA Materials Studio. http://accelrys.com/products/collaborative-science/biovia-materials-studio/.

[25] Koelling D.D., Harmon B.N. (1977). Technique for relativistic spin-polarized calculations. J. Phys. C Solid State Phys..

[26] Perdew J.P., Burke K., Ernzerhof M. (1996). Generalized Gradient Approximation Made Simple. M. Phys. Rev. Lett..

[27] Tkatchenko A., Scheffler M. (2009). Accurate Molecular Van Der Waals Interactions from Ground-State Electron Density and Free-Atom Reference Data. M. Phys. Rev. Lett..

[28] Perdew J.P., Chevary J.A., Vosko S.H., Jackson K.A., Pederson M.R., Singh D.J., Fiolhais C. (1992). Atoms, molecules, solids, and surfaces: Applications of the generalized gradient approximation for exchange and correlation. Phys. Rev. B.

[29] Ortmann F., Bechstedt F., Schmidt W.G. (2006). Semiempirical van der Waals correction to the density functional description of solids and molecular structures. Phys. Rev. B.

[30] Hammer B., Hansen L.B., Norskov J.K. (1999). Improved adsorption energetics within density-functional theory using revised Perdew-Burke-Ernzerhof functionals. Phys. Rev. B.

[31] Wu Z., Cohen R.E. (2006). More accurate generalized gradient approximation for solids. Phys. Rev. B.

[32] Perdew J.P., Ruzsinszky A., Csonka G.I., Vydrov O.A., Scuseria G.E., Constantin L.A., Zhou X., Burke K. (2008). Restoring the Density-Gradient Expansion for Exchange in Solids and Surfaces. Phys. Rev. Lett..

[33] Perdew J.P., Zunger A. (1981). Self-interaction correction to density-functional approximations for many-electron systems. Phys. Rev. B.

[34] Ceperley D.M., Alder B.J. (1980). Ground State of the Electron Gas by a Stochastic Method. Phys. Rev. Lett..

[35] Pfrommer B.G., Cote M., Louie S.G., Cohen M.L. (1997). Relaxation of Crystals with the Quasi-Newton Method. J. Comput. Phys..

[36] Monkhorst H.J., Pack J.D. (1977). “Special points for Brillouin-zone integrations”—A reply. Phys. Rev. B.

[37] Alfe D. (1999). Ab initio molecular dynamics: Analytically continued energy functionals and insights into iterative solutions. Comput. Phys. Commun..

[38] Colmenero F., Bonales L.J., Cobos J., Timón V. (2017). Inelastic and Reactive Scattering Dynamics of Hyperthermal O and O_2_ on Hot Vitreous Carbon Surfaces. J. Phys. Chem. C.

